# Preoperative Cachexia as a Predictor of Postoperative Morbidity and a Target for Home-Based Prehabilitation in Resectable Gastric Cancer

**DOI:** 10.3390/cancers18020324

**Published:** 2026-01-20

**Authors:** Vladimir Konstantinovich Lyadov, Tatiana Sergeevna Boldyreva, Alexander Yuryevich Gorshkov, Elena Vitalievna Zyatenkova, Anna Yurievna Ikonnikova, Mikhail Georgievich Chashchin, Vsevolod Nikolaevich Galkin

**Affiliations:** 1 Russian Medical Academy of Continuous Professional Education, 125993 Moscow, Russia; 2 Moscow State Budgetary Healthcare Institution «Moscow City Hospital Named After S.S. Yudin, Moscow Healthcare Department», 117152 Moscow, Russia; 3 Department of Oncology, Novokuznetsk State Institute for Postgraduate Medical Education, 654005 Novokuznetsk, Russia; 4 National Medical Research Center for Therapy and Preventive Medicine, 101990 Moscow, Russia; 5 Department of Therapy and Preventive Medicine, Yevdokimov Moscow State University of Medicine and Dentistry, 127473 Moscow, Russia; 6 Laboratory of Biological Microchips, Engelhardt Institute of Molecular Biology, Russian Academy of Sciences, 119991 Moscow, Russia

**Keywords:** prehabilitation, gastric cancer, cachexia, sarcopenia

## Abstract

**Background:** Gastric cancer (GC) is one of the most common malignancies, requires aggressive treatment, as has a high incidence of complications. The high prevalence of cachexia and comorbidity among GC patients has led to the development of the “prehabilitation” concept. We aimed to investigate the prognostic value of cachexia in the “Western” patient population with resectable GC and to evaluate its utility as an indicator for a home-based prehabilitation program. **Methods:** This cohort study included 147 patients who underwent surgical treatment for GC from 2019 to 2023. A multivariable analysis was conducted to study the impact of cachexia on postoperative outcomes in 122 patients with resectable GC. The prehabilitation group included 25 patients with cachexia who underwent a 2-week-long multimodal prehabilitation program prior to surgery. The functional results, as well as the 30-day incidence of postoperative complications and 90-day mortality, were evaluated. **Results:** There were 76 (51.7%) patients with cachexia. Multivariate analysis revealed that cachexia was a significant predictor of all postoperative complications (OR = 5.48, 95% CI 1.85–18.39, *p* = 0.001), severe postoperative complications (OR = 15.87, 95% CI 3.05–131.81, *p* < 0.001) and surgical site infection (SSI) (OR = 8.03, 95% CI 1.89–49.09, *p* = 0.038). Patients in the prehabilitation group had a lower incidence of SSI than in the control group (8.3% vs. 23.5%, *p* = 0.049). **Conclusions:** Preoperative cachexia is a potentially modifiable predictor of complications after gastric cancer surgery, and its identification may help define high-risk patients for proactive multimodal prehabilitation.

## Simple Summary

Gastric cancer surgery is complex, and identifying potential adverse factors of surgical treatment is essential to prevent complications and improve outcomes. This study investigated the prevalence and impact of cachexia on short-term outcomes of surgical treatment in patients with resectable gastric cancer and evaluated the safety and effectiveness of comprehensive multimodal prehabilitation in this group of patients. Cachexia was associated with an increased incidence of postoperative complications, including surgical site infections. Multimodal prehabilitation improved functional capacity and promoted weight gain in patients with gastric cancer and cachexia. Furthermore, the implementation of multimodal prehabilitation was associated with a lower incidence of surgical site infections. These findings suggest that cachexia is an important predictor in patients with resectable gastric cancer. Prehabilitation has the potential to improve functional capacity and postoperative outcomes in cachectic patients with resectable gastric cancer.

## 1. Introduction

Gastric cancer (GC) is the fifth most common cancer in terms of both incidence (4.8%, 968,784 new cases) and mortality (6.8%, 660,175 deaths) among all types of malignancy [1]. Surgery is the key treatment modality in patients with localized or locally advanced disease. Unfortunately, the incidence of postoperative complications and mortality after gastrectomy remains high, especially in the “Western” patient population [2].

Cachexia was shown to impair quality of life, worsen response to treatment, and decrease the likelihood of survival in patients with various malignancies [3,4]. The European Palliative Care Research Collaboration (EPCRC)’s definition of cancer cachexia allows for the diagnosis of cachexia even in cases of minimal weight loss (5% or 2% in the presence of sarcopenia or BMI < 20 kg/m^2^) [5,6]. A recent meta-analysis including 7186 patients with esophageal and gastric cancer not only showed cachexia to be present in 35% of patients but also proved its pronounced negative impact on overall survival (OS) (OR = 1.46, 95% CI: 1.31–1.63, *p* < 0.001) [3]. However, there is a lack of research evaluating the prognostic value of cachexia in relation to postoperative complications in patients with resectable GC. We aimed to evaluate the prevalence of cancer cachexia in patients with resectable GC and its correlation with surgical morbidity.

It is important to note that patients with GC often seek medical care due to the development of tumor-related complications, such as bleeding, gastric outlet obstruction, or other complications, which frequently results in delayed diagnosis and the initiation of the cachexia process. Regardless, the first critical milestone is establishing the diagnosis. Only after this can the multidisciplinary team convene to define the diagnostic pathway, while a nutritionist takes responsibility for optimizing the patient’s condition, ensuring he or she is in the best possible state for surgery whenever feasible [7].

Advanced age, co-morbidity, malnutrition, cachexia, low functional status, and negative effects of neoadjuvant chemotherapy in patients with GC all lead to the gradual adoption of the “prehabilitation” concept, which implies proactive preparation of patients for surgery by means of exercise therapy and nutritional and psychological interventions [8]. Several studies have been published assessing the safety and efficiency of multimodal prehabilitation in patients with gastric cancer [9,10]. However, the target cohort of participants in prehabilitation programs is yet to be determined, with older age and frailty being evident indications. For instance, Chen et al. [11], in a randomized controlled setting, showed that the rate of severe complications significantly decreased after a 3-week multimodal prehabilitation program in elderly (>65 years) frail gastric cancer patients (11.1% vs. 25.9%, *p* = 0.04).

Major obstacles to the implementation of multimodal prehabilitation into routine clinical practice include the lack of commonly accepted indications, the risk of treatment delay, and the immense effort needed to introduce such a program into a standardized surgical pathway. Thus, we conducted a feasibility trial of a brief (2-week) home-based prehabilitation program in patients with resectable GC and cachexia.

## 2. Materials and Methods

### 2.1. Characteristics of Patients

The prevalence of cancer cachexia was analyzed in a prospective cohort of 147 patients with resectable gastric cancer who underwent surgical treatment at the Department of Oncology No. 4 of Moscow City Hospital named after S.S. Yudin, Moscow Healthcare Department from 2019 to 2023. The study was conducted in two parts.

Part 1 assessed the impact of cachexia on short-term surgical outcomes in 122 patients with resectable gastric cancer who underwent surgery between 2019 and 2022, prior to the implementation of a prehabilitation program. All patients who received surgical treatment were included.

**Figure 1:**
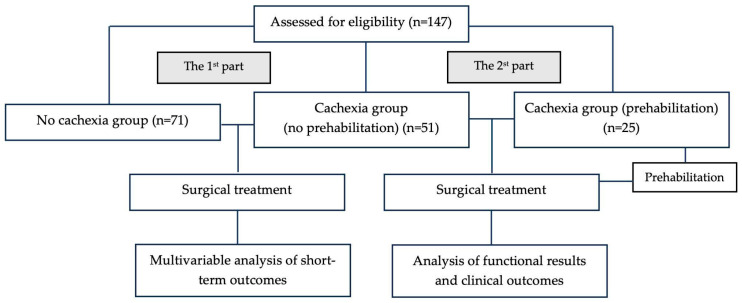
Schematic overview of the study.

Part 2 was a pilot comparative study evaluating the safety and efficacy of multimodal remote prehabilitation in patients with cachexia preparing for surgical treatment of gastric cancer between 2022 and 2023. The prehabilitation group included 25 patients. The control group included all patients with cachexia from the first part of the study who underwent radical gastrectomy without prehabilitation (Figure 1).

Only patients with histologically confirmed stage I-III gastric cancer were included. The study was approved by the Local Ethics Committee of the Russian Medical Academy of Continuous Professional Education, protocol No. 6, dated 30 May 2022. Informed consent was obtained from all the participating patients. Comorbidity was classified according to the Charlson comorbidity index (CCI) [12]. The main demographic and clinical data of patients are presented in Table 1.

### 2.2. Assessment of Cachexia and Sarcopenia

The presence of cancer cachexia was established according to EPCRC criteria: weight loss > 5% in 6 months in the absence of fasting and/or weight loss > 2% and BMI < 20 kg/m^2^ and/or weight loss > 2% and the presence of sarcopenia.

Confirmed sarcopenia was defined according to the European Working Group on Sarcopenia in Older People 2 (EWGSOP2) as the presence of low muscle strength and low SMI [6]. Muscle strength was assessed by measuring handgrip strength using an electronic dynamometer (DMER-150-0.5 wrist electronic dynamometer, “TVES”, Tula, Russia) within three days prior to surgery. Two trials were conducted for each hand (dominant and nondominant), with a one-minute rest interval between attempts, and the highest value was used for analysis. Low muscle strength was defined as <27 kg for men and <16 kg for women [13]. Skeletal muscle mass was estimated from the axial section of a CT scan at the level of the third lumbar vertebra (L3) using the specialized software SliceOmatic, ver. 6 (TomoVision, Montreal, QC, Canada). Skeletal muscle index (SMI) was calculated as the ratio of lumbar skeletal muscle area to height squared (cm^2^/m^2^). The SMI cut-offs (43 and 53 cm^2^/m^2^ in men with a BMI < and >25 kg/m^2^, respectively, and 41 cm^2^/m^2^ in women) suggested by Martin et al. for “Western-type” patients with different body weights were used to assess the presence of sarcopenia [14]. Physical performance was assessed within 3 days before surgery using the 400 m walking test (400MWT), with each patient asked to complete 20 laps of 20 m. The 6 min cut-off was used to diagnose low physical capacity and was necessary for the diagnosis of severe sarcopenia [15].

Cardiopulmonary exercise testing (CPET) using a bicycle ergometer was used to evaluate patients’ tolerance to exercise training in metabolic equivalents of task (METs) (modified Bruce protocol) [16].

### 2.3. Multimodal Prehabilitation

We developed a 2-week home-based multimodal prehabilitation program starting immediately after surgical staging (diagnostic laparoscopy) or the completion of neoadjuvant therapy. During the first visit, patients were consulted by a kinesiologist to determine the frequency and duration of exercise training based on their level of initial functional capacity. The exercise program included 5–10 min of standing and breathing exercises, 20–40 min of aerobic exercise via Nordic walking, and 5–10 min of stretching exercises. We also provided the patients with educational videos that included all the exercises to be repeated at home.

According to World Health Organization (WHO) guidelines, patients were prescribed at least 600 MET minutes per week (150 min of moderate-intensity activity), with the aim of increasing this to 1200 MET minutes per week (300 min of moderate-intensity activity per week) [17]. The intensity of aerobic exercise was personalized based on the target heart rate (HR) and the Borg rating of perceived exertion scale [18]. The program was aimed at achieving moderate- or high-intensity training, corresponding to an RPE from 13 to 16, and maintaining a target HR, which was calculated using the Karvonen formula: (220 − age − resting HR) × 70% + resting HR [19]. Patients were trained at home under the supervision of a physical therapist using telephone control and/or a fitness tracker, which transmitted information about their HR to the therapist during training. In addition, patients recorded the frequency, duration, and intensity of their training in exercise diaries.

Patients in the prehabilitation group were asked to keep daily food diaries and receive additional therapeutic nutrition (Supportan drink^®^, Fresenius Kabi, Bad Homburg, Germany, 200 mL) based on their needs, total caloric intake (30 kcal per 1 kg of body weight per day), and daily protein requirement (1.5 g per kg of body weight per day).

Before starting prehabilitation, patients received a consultation with a clinical psychologist to assess their initial condition using the Hospital Anxiety and Depression Scale (HADS) and the Spielberger–Khanin anxiety test. They were repeatedly consulted by the psychologist in the middle of the program and immediately before surgery.

Patients were reassessed at the end of prehabilitation, on the day of their admission for surgery.

### 2.4. Surgical Treatment

All the patients underwent radical gastrectomy with D2 lymph node dissection, depending on the location of the tumor. Multivisceral resections could be performed in case of adjacent organ involvement. D1 + lymph node dissection was accepted in early gastric cancer. Omentectomy was performed in cT3-4 tumors. Roux-en-Y reconstruction was typically performed. After proximal gastrectomy, a double-tract reconstruction was carried out.

A standardized perioperative management protocol (ERAS) was applied in all the patients: antibiotic prophylaxis, thromboprophylaxis, avoiding bowel preparation, and prolonged fasting. The intraoperative placement of a nasointestinal feeding tube was not routinely performed. Regardless of study group allocation, all the patients were mobilized on the first postoperative day, were allowed oral intake of water, and received supplemental oral nutritional support of up to 500 mL per day (Supportan drink^®^, Fresenius Kabi, 200 mL). On the 3rd day after surgery, if there was no significant elevation in inflammatory blood markers (C-reactive protein, leucocyte level), the volume of enteral nutrition was gradually increased.

### 2.5. Study Outcomes

The primary outcome was the number of 30-day postoperative complications according to the Clavien–Dindo classification. All complications were graded I–V according to the Clavien–Dindo classification. Mild postoperative complications (Clavien–Dindo I–II) were defined as events not requiring invasive treatment, while severe complications included all complications that required invasive manipulations for their treatment (Clavien–Dindo ≥ IIIa).

Other clinical outcomes included 30- and 90-day mortality, as well as the incidence of surgical site infection (SSI) and anastomotic leakage. Surgical site infection (SSI) was defined according to the Centers for Disease Control and Prevention (CDC criteria) as an infection occurring at or near the surgical incision within 30 days after the procedure [20]. SSI included superficial incisional, deep incisional, or organ/space, based on the depth of tissue involvement and clinical or microbiological evidence. SSI was characterized by abdominal symptoms and clinical signs, including pain, tenderness, and rebound tenderness, as well as by supporting evidence from imaging studies (e.g., intra-abdominal abscess) or positive bacterial cultures from intra-abdominal swabs or drainage fluid. The diagnosis of specific postoperative surgical complications, including anastomotic failure, duodenal stump leak, and pancreatic fistula, was established in accordance with the International consensus on a complications list after gastrectomy for cancer [21].

Functional and nutritional outcomes for the prehabilitation group included 400 m walking speed, handgrip strength dynamics, weight, body mass index (BMI), albumin level, and neutrophil-to-lymphocyte ratio (NLR).

### 2.6. Statistical Analysis

The normality of the data was assessed visually and using the Kolmogorov–Smirnov and Shapiro–Wilk normality tests. Depending on their distribution, continuous variables are presented as either mean ± standard deviation or median [interquartile range, IQR]. Qualitative variables were expressed in numbers and percentages (*n*, %). The Student’s *t*-test was used to compare parametric data, while the Mann–Whitney U-test was used to compare non-parametric data (for independent samples) or the Wilcoxon signed-rank test (for dependent samples). Categorical variables were compared using Fisher’s exact test. McNemar’s test was used on paired nominal data.

Univariable logistic regression analysis was performed to calculate the odds ratios (ORs) and the 95% confidence interval (95% CI) between factors and short-term outcomes. Subsequently, a multivariable analysis was conducted using Firth’s penalized regression. We evaluated multicollinearity among candidate variables using the variance inflation factor (VIF). Variables with VIF values exceeding 5 were considered for removal based on collinearity. Model discrimination was evaluated using the receiver operating characteristic (ROC) curve, with the area under the curve (AUC) quantifying performance. Model calibration was assessed using the calibration-in-the-large method. Calibration was deemed adequate when the absolute difference between the mean predicted probability and the observed event rate was less than 0.05.

A *p*-Value less than 0.05 was considered statistically significant. Statistical analysis was performed using Jamovi version 1.6 (Australia) and R version 4.3.3 (R Foundation for Statistical Computing, Vienna, Austria), using the packages “logistf”, “car”, and “pROC”.

All statistical analyses adhered to the STROBE recommendations for cohort studies.

## 3. Results

### 3.1. The Prevalence of Cachexia

The prevalence of cachexia was assessed in 147 patients prior to surgical treatment for resectable gastric cancer. Weight loss greater than 5% in the last 6 months was detected in 72 patients. Weight loss greater than 2% was observed in 4 patients with sarcopenia or a BMI < 20 kg/m^2^. Thus, cachexia was diagnosed in 76 patients (51.7%) (Table 2).

Cachexia was detected in 53.1% of patients with stage III cancer and in 34.6% of patients (*p* = 0.044) with stage I-II cancer. Patients with cachexia were in the worst condition at the time of diagnosis (ECOG-2 status 29.4% vs. ECOG 0–1 9.9%, *p* = 0.006) and were also more likely to undergo perioperative chemotherapy using the FOLFOX regimen (23.6% vs. 8.4%, *p* = 0.021). Also, gastric outlet obstruction and bleeding were more often diagnosed in the cachexia group than in the control group (9.8% vs. 0%, *p* = 0.028; 5.8% vs. 0%, *p* = 0.039).

### 3.2. Impact of Cachexia on Postoperative Morbidity and Mortality

Table 3 shows the postoperative results depending on the presence of cachexia.

The following factors were used for univariable analysis: age, gender, Charlson comorbidity index (CCI), neoadjuvant chemotherapy (NACT), cachexia, stage, ECOG status, laparoscopic approach, and type of surgery (Table 4). On multivariable analysis, cachexia (OR = 5.48, 95% CI 1.85–18.39, *p* = 0.001) and male sex (OR = 4.39, 95% CI 1.4–16.9, *p* = 0.009) were significant risk factors for all postoperative complications. Cachexia (OR = 15.87, 95% CI 3.05–131.81, *p* < 0.001) and male sex (OR = 9.74, 95% CI 1.79–110.23, *p* = 0.005) also appeared to predict severe postoperative complications (Clavien–Dindo ≥ IIIa), whereas NACT was associated with a lower incidence of severe postoperative complications (OR = 0.11, 95% CI 0.02–0.47, *p* = 0.002). In addition, cachexia was the only independent negative predictor for SSI (OR = 8.03, 95% CI 1.89–49.09, *p* = 0.038) (Table 4).

All models demonstrated strong discrimination (AUC for all complications = 0.844, 95% CI 0.749–0.938; AUC for severe complications = 0.927, 95% CI 0.868–0.987; AUC for SSI = 0.863, 95% CI 0.722–1). The model diagnostics demonstrated good calibration for all models and the absence of multicollinearity (Supplementary Tables S1 and S2).

### 3.3. Results of Prehabilitation

**Figure 2:**
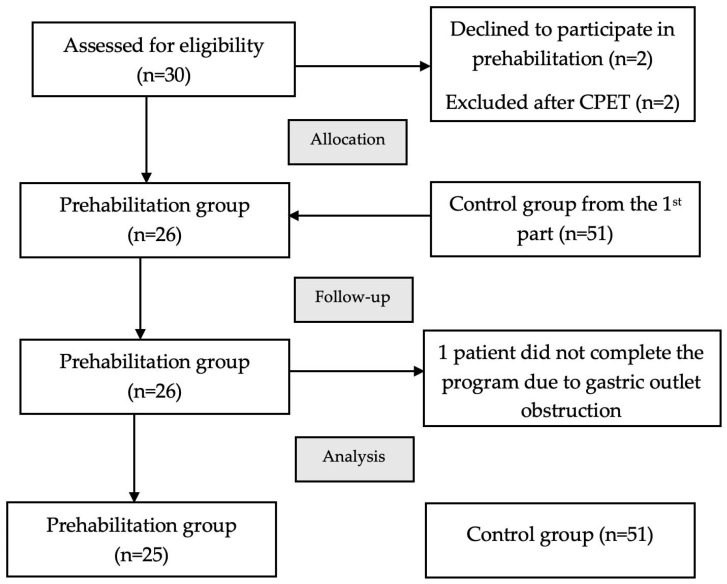
CONSORT flow diagram. Flowchart of part 2 of the study. CPET—cardiopulmonary exercise testing.

Thirty patients with resectable gastric cancer and cachexia were included in the feasibility prehabilitation program. Four patients were excluded: two declined to participate in the program after initial agreement and two had ischemic changes on their electrocardiograms during CPET. Additionally, one patient was unable to complete the program due to a decompensated gastric outlet obstruction. Consequently, twenty-five patients were included in the prehabilitation outcomes analysis. Figure 2 illustrates the flow diagram according to the CONSORT guidelines, including reasons for participant dropout.

No statistically significant differences were found between groups for any of the characteristics (*p* > 0.05) (Supplementary Table S3).

The duration of prehabilitation ranged from 9 days to 21 days, with a median of 15 days. The completion rate was 100%. One patient underwent inpatient prehabilitation because of an abscess caused by direct tumor invasion of the liver. One patient developed deep vein thrombosis (DVT) at the end of prehabilitation, which delayed his surgery.

### 3.4. Effects of Prehabilitation on Postoperative Results

The postoperative results by group are presented in Table 5.

The SSI rate was significantly lower in the prehabilitation group (8.3% vs. 23.5%, *p* = 0.049). For other outcomes (all complications, severe complications, anastomotic leakage, and pneumonia), the differences did not reach statistical significance.

### 3.5. Effects of Prehabilitation on Functional and Nutritional Status

The dynamics of functional and nutritional status of the prehabilitation group are shown in Table 6. After prehabilitation, 9 (36%) of the patients had increased physical capacity measured in MET (*p* = 0.008). Weight gain was present in 17 patients (0.5–6 kg, *p* < 0.001), and 1 patient’s weight decreased by 1 kg.

## 4. Discussion

Comorbidity, anemia, and cachexia are often present in GC patients. The coexistence of these adverse factors rarely precludes surgery. This can lead to an increased risk of postoperative complications and mortality. Additionally, the deleterious effects of the neoadjuvant treatment may further affect surgical outcomes [22]. Pasquer et al. analyzed the results of more than 7900 gastrectomies and showed that the 30-day mortality rate in elderly patients with multiple comorbidities (CCI ≥ 3) exceeded 10% even in high-volume centers [23]. At the same time, numerous studies have found that mortality and the frequency of postoperative complications are higher for comorbid patients with malnutrition [2,24].

Cachexia has been shown to be a significant negative prognostic factor for overall survival in patients with resectable GC [3,4]. Unfortunately, data on the impact of cachexia on immediate postoperative outcomes following GC surgery remain limited and heterogeneous. For example, Zhang et al. reported that cachexia diagnosed according to the Asian Working Group for Cachexia (AWGC-defined cachexia) was a significant independent predictor of postoperative complications in Asian patients with curable GC, whereas Fearon’s criteria were not [25]. Additionally, a prospective randomized controlled trial conducted in China evaluated 112 GC cachexia patients and reported improved short-term postoperative outcomes with preoperative immunonutrition, particularly with a reduction in overall incidence of complications (28.6% vs. 44.6%, *p* = 0.049) and infectious complications (21.4% vs. 37.5%, *p* = 0.040), which corresponds well with the results of our study [26]. Accordingly, we aimed to determine the prevalence of cachexia according to the Fearon international consensus criteria in a “Western” patient population. An additional objective was to evaluate the feasibility and clinical utility of multimodal prehabilitation in patients with resectable GC in the presence of cachexia.

Our study shows that cachexia defined according to EPCRC criteria is highly prevalent in “Western” patients with potentially resectable GC and contributes to an increased risk of postoperative complications. In addition, this study performed a comprehensive preoperative assessment of sarcopenia in patients with GC. It should be noted that in the vast majority of studies investigating the impact of cachexia or sarcopenia on treatment outcomes in patients with various malignancies, sarcopenia is diagnosed solely on the basis of low muscle mass, which contradicts the current consensus definition of sarcopenia [6,26,27]. In the present study, we applied comprehensive diagnostic criteria requiring the assessment of both muscle strength and skeletal muscle mass to confirm the diagnosis of sarcopenia. As a result, only a small proportion of patients in the study cohort met the internationally acknowledged criteria for confirmed sarcopenia (1.36%). Given the limited number of patients in the subgroup “cachexia associated with low muscle mass/strength”, a sensitivity analysis comparing “weight-loss-only cachexia” with “cachexia associated with low muscle mass/strength” was not performed. In our view, these findings may reflect the multifactorial nature of sarcopenia and the limitations of current diagnostic criteria.

Cachexia was shown to be a significant independent risk factor for postoperative complications, severe postoperative complications, and SSI in a multivariable analysis. Moreover, male gender was also associated with an increased risk of overall and severe postoperative complications, which perfectly corresponds to the results of a major multicenter study including 9351 patients that demonstrated male patients suffered more overall and major complications, including anastomotic leakages and pneumonia [28]. In our study, NACT was associated with a lower incidence of severe postoperative complications, which also aligns with some population-based analyses and meta-analyses that demonstrated comparable or less significant surgical morbidity in patients who had a gastrectomy after NACT [29,30]. Despite these findings, further studies are required to confirm and expand these results.

The impact of structured preoperative preparation (prehabilitation) on surgical outcomes in patients with gastric cancer has been a topic of debate for a long time. In 2019, Bolger et al. [31] conducted a systematic review of the literature to evaluate the effectiveness of prehabilitation prior to surgery in patients with esophageal and gastric cancer. The study included 12 articles that summarized the treatment outcomes of 937 patients. An extreme heterogeneity of the studies was mentioned, with most authors focusing on the effects of physical therapy, nutritional support, or respiratory exercise programs separately, thus making it difficult to conduct a meta-analysis and determine the overall impact of prehabilitation on gastric cancer treatment. Only the study by Yamamoto et al. included both physical and nutritional interventions and showed the improvement of functional parameters such as hand grip strength, which increased from 20.0 ± 5.3 kg to 21.2 ± 5.2 kg (*p* = 0.022). However, the lack of a control group and small sample size made it difficult to determine the impact of multimodal training on clinical outcomes [32].

Later, Tukanova et al. presented the results of a meta-analysis evaluating the results of perioperative physical therapy in patients treated by esophagectomy or gastrectomy [33]. They included 14 studies that summarized the prehabilitation outcomes of 960 patients. The meta-analysis showed a statistically significant reduction in the incidence of pneumonia (OR = 0.70, 95% CI 0.51–0.95, *p* = 0.02) and all postoperative complications (risk difference = −0.16, 95% CI −0.24–−0.09, *p* < 0.0001) in the prehabilitation group. However, prehabilitation was applied to all patients, regardless of their age or the presence of other medical conditions. In our view, prehabilitation is a time- and resource-consuming technology that should be preferably applied to those patients with a higher risk of postoperative complications.

Our study is probably the first to investigate the relationship between prehabilitation and postoperative results in gastric cancer patients with cachexia. It shows that a 2-week-long combination of home-based exercise and nutritional and psychological support can improve patients’ functional status and body composition even in the presence of cachexia. Furthermore, this study demonstrates the feasibility of the program in patients with cachexia, highlights their high level of adherence, and suggests a potential positive impact on clinical outcomes. The absence of clinically significant adverse events supports the safety and suitability of the program for cachexic patients, who are characterized by their frailty and functional decline. The completion rate was 96%, which is consistent with previous reports [33,34].

Finally, we support the concept of a home-based prehabilitation program that measures physical activity and nutrient consumption via activity trackers, diaries, and regular phone calls from the attending physician. In our opinion, this approach, along with the use of instructional videos, can enhance patients’ adherence to these programs.

All surgical procedures in the comparative groups were performed by the same surgical team, thereby minimizing the impact of potential confounding factors such as progression along the laparoscopic learning curve or refinement of surgical techniques on the study outcomes. The groups were constructed to be comparable in key baseline characteristics in order to minimize selection bias, and statistical analysis confirmed that there were no significant differences between the groups.

There are several limitations to this study. First, the second part of our study was a pilot study with a small number of participants, which limited our ability to detect statistically significant differences between groups in clinical outcomes. Additionally, the control group was a retrospective one, which allowed us to fully test functional parameters in only 54 patients. A small sample size and short follow-up period (median 25.5 months) precluded us from assessing the long-term impact of prehabilitation. Also, the cost-efficiency analysis of home-based multimodal prehabilitation was performed separately.

In this study, postoperative complications were classified according to the Clavien–Dindo classification system. Given the well-recognized limitations of this classification, an additional, separate analysis was performed for the most clinically significant complications in gastric surgery, namely SSI and anastomotic leakage.

Nevertheless, our data suggest that cachexia has a negative impact on patients with resectable gastric cancer. We also showed that comprehensive home-based prehabilitation might improve gastric cancer treatment outcomes in patients with cachexia. However, there are several important and unresolved issues regarding the optimal duration of these programs and their content, the feasibility of remote prehabilitation, and its indications and contraindications. Our findings justify the design of a multicenter RCT to determine whether targeted prehabilitation for cachectic patients can improve survival and reduce postoperative morbidity in gastric cancer. Finally, our study stresses the importance of pre-operative cachexia evaluation and possible correction in routine clinical practice.

## 5. Conclusions

Cachexia can be considered a negative predictor of short-term postoperative outcomes in patients with GC. Considering this during the shared decision-making process offers the potential for better risk stratification, targeted interventions, and, ultimately, improved outcomes. The effectiveness of multimodal home-based prehabilitation programs remains poorly understood, especially in patients with cachexia. This pilot study demonstrated the feasibility and safety of multimodal training in patients with resectable GC and cachexia. The implementation of such programs, following their evaluation in multicenter studies, may become an important tool for improving surgical outcomes in patients with GC, particularly in the presence of cachexia, by increasing functional reserves and reducing postoperative complications.

## Figures and Tables

**Table 1:** Clinical characteristics of patients.

Characteristics	Result (*n* = 147)
Male, *n* (%)	92 (62.6)
Age in years, median (interquartile range)	67 (61–74)
ECOG status (score), *n* (%)	
0–1	121 (82.3)
2	26 (17.6)
CCI (score), median (interquartile range)	5 (4–6)
Complications of cancer, *n* (%)	
Gastric outlet obstruction	5 (3.4)
Bleeding	5 (3.4)
Anemia	30 (20.4)
Histological type, *n* (%)	
Adenocarcinoma low grade	82 (55.8)
Adenocarcinoma high grade	47 (31.9)
SRCC	18 (12.2)
Surgical procedures, *n* (%)	
Proximal gastrectomy	7 (4.8)
Total gastrectomy	55 (37.6)
Distal gastrectomy	75 (51.3)
Combined	9 (6.1)
Laparoscopic surgery, *n* (%)	57 (39)
Pathomorphological stage (yp/*p*), *n* (%)	
Complete response	14 (9.5)
I	50 (34.2)
II	33 (22.6)
III	49 (33.5)
Perioperative chemotherapy, *n* (%)	
FLOT	58 (39.7)
FOLFOX	25 (17.1)
Adjuvant chemotherapy, *n* (%)	12 (8.2)

CCI—Charlson comorbidity index, SRCC—signet ring cell carcinoma.

**Table 2:** Body composition and nutritional status of patients.

Characteristics	Result (*n* = 147)
Cachexia, *n* (%)	76 (51.7)
Body weight loss in the last 6 months (%), median (interquartile range)	3.4 (0–36)
NRS-2002 ≥ 3 (score), *n* (%)	63 (42.8)
BMI (kg/m^2^), median (interquartile range)	26.5 (23.3–29.5)
NLR ≥ 3, *n* (%)	33 (22.4)
Albumin < 35 (g/L), *n* (%)	21 (14.3)
SMI (cm^2^/m^2^), male	53.1 ± 10.8
SMI (cm^2^/m^2^), female	44.8 ± 9.67
Sarcopenia (Martin criteria + low muscle strength), *n* (%)	2 (1.36)

BMI—body mass index, NRS—Nutritional Risk Screening 2002, SMI—skeletal mass index, NLR—neutrophil-to-lymphocyte ratio.

**Table 3:** Incidence of postoperative events depending on the presence of cachexia.

Results	All Patients (*n* = 122)	Cachexia (*n* = 51)	No Cachexia (*n* = 71)	*p*-Value
All complications (Clavien–Dindo I-V)	29 (23.7)	20 (39.2)	9 (12.6)	<0.001 *
Mild complications (Clavien–Dindo I-II)	11 (9)	7(13.7)	4 (5.6)	0.198 *
Severe complications (Clavien–Dindo ≥ IIIa)	18 (14.7)	13 (25.4)	5 (7)	0.008 *
Anastomotic leakage	5 (4)	5 (9.8)	0	0.011 *
SSI	14 (11.4)	12 (23.5)	2 (2.8)	<0.001 *
30-day mortality	4 (3.2)	4 (7.8)	0	0.027 *
90-day mortality	6 (4.9)	5 (9.8)	1 (1.4)	0.081 *

SSI—surgical site infection; qualitative variables are expressed in numbers and percentages (*n*, %); * Fisher’s exact test.

**Table 4:** Factors associated with postoperative complications.

Factors	Events (Yes/No)	Univariable Analysis			Multivariable Analysis		
		OR	95% CI	*p*-Value	OR	95% CI	*p*-Value
All postoperative complications (Clavien-Dindo I-V) (*n* = 29)							
Gender							
Female	5/37						
Male	24/56	2.96	1.14–8.94	0.024	4.39	1.4–16.9	0.009
Age		1.08	1.03–1.14	0.001	1.02	0.94–1.12	0.619
NACT							
No	15/33						
Yes	14/60	1.13	0.52–2.46	0.747	0.65	0.22–1.86	0.424
CCI		1.82	1.32–2.63	<0.001	1.68	0.92–3.12	0.091
ECOG							
0–1	20/80						
2	9/13	2.76	1.04–7.22	0.042	1.72	0.46–6.51	0.42
Cachexia							
No	9/62						
Yes	20/31	4.28	1.82–10.72	<0.001	5.48	1.85–18.39	0.001
Stage III							
No	19/64						
Yes	10/29	1.18	0.48–2.77	0.713	0.6	0.2–1.66	0.331
Laparoscopic approach							
No	17/58						
Yes	12/35	1.18	0.5–2.71	0.703	1.48	0.51–4.41	0.473
Gastrectomy							
No	19/57						
Yes	10/36	0.85	0.35–1.97	0.705	0.85	0.27–2.59	0.773
Severe complications (Clavien–Dindo ≥ IIIa) (*n* = 18)							
Gender							
Female	2/40						
Male	16/64	4.14	1.21–21.65	0.022	9.74	1.79–110.23	0.005
Age		1.10	1.03–1.18	0.002	0.99	0.87–1.13	0.819
NACT							
No	14/34						
Yes	4/70	0.15	0.04–0.44	<0.001	0.11	0.02–0.47	0.002
CCI		2.03	1.38–3.18	<0.001	2.07	0.88–5.05	0.096
ECOG							
0–1	12/88						
2	6/16	2.79	0.9–8.12	0.073	1.71	0.27–12.36	0.565
Cachexia							
No	5/66						
Yes	13/38	4.24	1.52–13.37	0.005	15.87	3.05–131.81	<0.001
Stage III							
No	11/72						
Yes	7/32	1.45	0.51–3.98	0.47	0.9	0.21–3.46	0.874
Laparoscopic approach							
No	8/67						
Yes	10/37	2.22	0.83–6.13	0.112	3.86	0.91–19.45	0.066
Gastrectomy							
No	13/63						
Yes	5/41	0.62	0.2–1.74	0.374	0.7	0.13–3.5	0.668
SSI (*n* = 14)							
Gender							
Female	3/39						
Male	11/69	1.87	0.58–7.68	0.311	1.9	0.48–9.47	0.369
Age		1.06	1–1.14	0.05	1.0	0.9–1.13	0.934
NACT							
No	8/40						
Yes	6/68	0.45	0.15–1.34	0.152	0.44	0.11–1.68	0.228
CCI		1.66	1.13–2.55	0.01	1.38	0.66–2.83	0.381
ECOG							
0–1	8/92						
2	6/26	4.29	1.32–13.57	0.016	2.13	0.49–9.57	0.309
Cachexia							
No	2/69						
Yes	12/39	8.80	2.47–46.76	<0.001	8.03	1.89–49.09	0.038
Stage III							
No	8/75						
Yes	6/33	1.72	0.55–5.17	0.337	1.08	0.29–3.82	0.902
Laparoscopic approach							
No	8/67						
Yes	6/41	1.24	0.4–3.7	0.696	1.86	0.48–7.74	0.368
Gastrectomy							
No	8/68						
Yes	6/40	1.29	0.42–3.85	0.646	1.29	0.33–5.07	0.713

CCI—Charlson comorbidity index, SSI—surgical site infection, NACT—neoadjuvant chemotherapy.

**Table 5:** Postoperative results depending on prehabilitation.

Results	Prehabilitation Group (*n* = 25)	Control Group (*n* = 51)	*p*-Value
All complications	5 (20)	20 (39.2)	0.079 *
Mild complications	2 (8)	7 (13.7)	0.709 *
Severe complications	3 (12)	13 (25,4)	0.238 *
SSI	1 (4)	12 (23.5)	0.049 *
Pneumonia	1 (4)	4 (7.8)	1.0 *
Anastomotic leakage	1 (4)	5 (9.8)	0.657 *
30-day mortality	0	4 (8)	0.297 *
90-day mortality	0	5 (10)	0.167 *
Length of stay (days)	9.5 (5–79)	10 (4–62)	0.482 **

SSI—surgical site infection; non-parametric data are displayed as median values (interquartile range); qualitative variables are expressed in numbers and percentages (*n*, %); * Fisher’s exact test; ** Mann–Whitney U-test.

**Table 6:** Dynamics of functional and nutritional status of patients before and after program.

Parameter	Before Prehabilitation	After Prehabilitation	*p*-Value
BMI (kg/m^2^)	24.4 ± 4.64	24.9 ± 4.88	<0.001 *
Body mass (kg)	65.1 ± 13.8	66.7 ± 14.1	<0.001 *
Handgrip strength (kg)	30.1 ± 8.36	31 ± 7.8	0.09 *
400MWT (min)	5.39 ± 1.01	5.28 ± 0.86	0.57 *
Physical capacity (MET)	3.45 ± 1.15	3.89 ± 1.04	0.008 *
Albumin (g/L)	37.3 ± 5.76	37.8 ± 5.13	0.39 *
NLR ≥ 3	3 (12)	5 (20)	0.41 **

BMI—body mass index, MET—metabolic equivalent of task, 400MWT—400 m walking test, NLR–neutrophil-to-lymphocyte ratio; * Student’s paired *t*-test; ** McNemar’s test; continuous parametric data are displayed as mean ± standard deviation; qualitative variables are expressed in numbers and percentages (*n*, %).

## Data Availability

The data presented in this study are available from the corresponding author upon reasonable request.

## Supplementary Materials

The following supporting information can be downloaded at https://www.mdpi.com/article/10.3390/cancers18020324/s1, Table S1: Model calibration for postoperative complications; Table S2: Variance inflation factors (VIF) for each variable; Table S3: Characteristics of patients depending on the study group.

## Abbreviations

The following abbreviations are used in this manuscript:

BMI	Body mass index
CCI	Charlson comorbidity index
CPET	Cardiopulmonary exercise testing
DVT	Deep vein thrombosis
GC	Gastric cancer
EPCRC	European Palliative Care Research Collaboration
EWGSOP2	European Working Group on Sarcopenia in Older People 2
HADS	The Hospital Anxiety and Depression Scale
HR	Heart rate
MET	Metabolic equivalent of task
NACT	Neoadjuvant chemotherapy
NLR	Neutrophil-to-lymphocyte ratio
NRS	Nutritional Risk Screening 2002
SMI	Skeletal muscle index
SSI	Surgical site infection
SRCC	Signet ring cell carcinoma
WHO	World Health Organization
400MWT	The 400 m walking test
