# Supplementary material for: Preoperative Cachexia as a Predictor of Postoperative Morbidity and a Target for Home-Based Prehabilitation in Resectable Gastric Cancer
Source: Cancers (Basel). 2026 Jan 20;18(2):324. doi: 10.3390/cancers18020324 (PMC12838876; doi:10.3390/cancers18020324)
Supplement: Supplementary file 1 [file cancers-18-00324-s001.zip › cancers-4084962-supplementary.pdf]

Supplementary Materials

Table S1. Model calibration for postoperative complications

Outcome	N	Events	Event rate, %	Mean predicted	Mean observed	Calibration error	Interpretation
**All complications**	122	29	23.7	0.251	0.238	0.013	Good (error < 0.05)
**Severe complications**	122	18	14.7	0.164	0.148	0.016	Good (error < 0.05)
**SSI**	122	14	11.4	0.135	0.115	0.021	Good (error < 0.05)

*SSI - surgical site infection

Table S2. Variance inflation factors (VIF) for each variable

	All complications	Severe complications	SSI
	**VIF**	**VIF**	**VIF**
**Cachexia**	1.47	2.31	1.27
**Age**	2.55	2.60	3.07
**Male**	1.19	1.41	1.15
**ECOG2**	1.36	1.79	1.40
**CCI**	2.39	2.71	2.78
**NACT**	1.25	1.83	1.32
**Laparoscopic approach**	1.24	1.49	1.29
**Stage III**	1.12	1.13	1.13
**Gastrectomy**	1.31	1.49	1.28

*CCI – Charlson comorbidity index, SSI - surgical site infection, NACT - neoadjuvant chemotherapy

Table S3. Characteristics of patients depending of the study group

Characteristics	Prehabilitation group (n=25)	Control group (n=51)	p-value
Male, *n* (%)	12 (48)	35 (68.6)	0.055
Age in years, median (interquartile range)	68 (63-75)	69 (61-75)	0.67
ECOG status (score), *n* (%)			
0-1	21 (84)	36 (70.6)	0.265
2	4 (16)	15 (29.4)	
CCI (score), median (interquartile range)	5 (4-7)	5 (4-6)	0.464
BMI (kg/m^2), median (interquartile range)	24.8 (21.3-28)	26 (22.8-28.6)	0.192
Histological type, *n* (%)			
Adenocarcinoma low grade	13 (52)	29 (56.8)	0.573
Adenocarcinoma high grade	9 (36)	18 (35.3)	
SRCC	3 (12)	4 (7.8)	0.667
Surgical procedures, *n* (%)			
Proximal gastrectomy	0	2 (3.9)	0.549
Total gastrectomy	9 (37.5)	22 (43.1)	0.731
Distal gastrectomy	11 (45.8)	24 (47)	0.743
Combined	4 (16.6)	3 (5.8)	0.2
Laparoscopic surgery, *n* (%)	10 (40)	15 (30)	0.356
Pathomorphological stage (yp/p), *n* (%)			
Complete response	1 (4.1)	7 (13.7)	0.259
I	6 (25)	13 (25.4)	0.964
II	7 (29.1)	7 (13.7)	0.11
III	10 (41.6)	24 (47)	0.561
Perioperative chemotherapy, n (%)			
FLOT	7 (29.1)	25 (49)	0.081
FOLFOX	7 (29.1)	12 (23.5)	0.672
Adjuvant chemotherapy, n (%)	3 (12.5)	1 (1.9)	0.09

*CCI – Charlson comorbidity index, SRCC – signet ring cell carcinoma, BMI - body mass index
